# Cross-cultural perception of strength, attractiveness, aggressiveness and helpfulness of Maasai male faces calibrated to handgrip strength

**DOI:** 10.1038/s41598-024-56607-z

**Published:** 2024-03-11

**Authors:** Marina L. Butovskaya, Victoria V. Rostovstseva, Anna A. Mezentseva, Alexander Kavina, Muhammad Rizwan, Yuanyuan Shi, Vítězslav Vilimek, Albert Davletshin

**Affiliations:** 1 Institute of Ethnology and Anthropology Russian Academy of Sciences, Leninsky Prospect 32a, 119334 Moscow, Russia; 2 National Research University, Higher School of Economics, Moscow, Russia; 3 St John’s University of Tanzania, Dodoma, Tanzania; 4 University of Haripur, Haripur, Pakistan; 5 Fudan University, Shanghai, China; 6 Ostrava University, Ostrava, Czech Republic; 7 Instituto de Antropología, Universidad Veracruzana, Xalapa, Mexico

**Keywords:** Geometric morphometrics, Hand grip strength, HGS, Composite portraits, Maasai, Strength, Attractiveness, Aggressiveness, Helpfulness, Perception, Cross-cultural ratings, Biological anthropology, Social anthropology, Behavioural ecology

## Abstract

Previous research has demonstrated that Maasai and Europeans tend to align in their ratings of the physical strength and aggressiveness of Maasai male faces, calibrated to hand grip strength (HGS). However, perceptions of attractiveness of these faces differed among populations. In this study, three morphs of young Maasai men created by means of geometric morphometrics, and depicting the average sample and two extrema (± 4 SD of HGS), were assessed by men and women from Tanzania, Czech Republic, Russia, Pakistan, China, and Mexico (total sample = 1540). The aim of this study was to test cross-cultural differences in the perception of young Maasai men’s composites calibrated to HGS, focusing on four traits: physical strength, attractiveness, aggressiveness, and helpfulness. Individuals from all six cultures were able to distinguish between low, medium, and high HGS portraits. Across all study populations, portrait of Maasai men with lower HGS was perceived as less attractive, more aggressive, and less helpful. This suggests that people from diverse populations share similar perceptions of physical strength based on facial shape, as well as attribute similar social qualities like aggressiveness and helpfulness to these facial images. Participants from all samples rated the composite image of weak Maasai men as the least attractive.

## Introduction

Initially, evolutionary anthropologists suggested that health and strength were crucial both for individual and group survival in ancestral humans^1,2^. Numerous studies have shown that hand grip strength (HGS) is a reliable indicator of muscularity and overall strength, including muscle strength in the upper arm, mid-upper arm, and calf circumferences, as well as skeletal muscle mass^3^. HGS has also been linked to ordinary gait speed^4^ and nutritional status^5^. Strength, and particularly HGS, could be an important object of accurate assessments (as indicator of superior physical attributes) in social encounters of early humans, and could be positively selected in human evolution both in men and women^6^. However, the marked sexual dimorphism in physical strength suggests the role of sexual selection with special accent on males, particularly in male–male physical competition^7^, protection from predators^8^, and hunting^9^. Besides, men displaying cues of physical strength are often perceived as more attractive partners by women^10^.

Men are universally stronger than women within a population, with even untrained men typically surpassing highly trained female athletes in HGS^11–14^. Grip strength tends to decline significantly with age in both men and women (^15^; though see:^13^). It was demonstrated that HGS is a marker of brain health and motor coordination quality^16^, suggesting the role of tool use and manufacturing in shaping precision and powerful gripping abilities during human evolutionary past^13,17^. Men had to be sensitive to strength-related features in possible rivals (male-male competition), and women may have developed a preference for strong men who could outcompete others, and had better chances for survival, as well as leaving stronger and healthier offspring (good-genes hypothesis)^18,19^. It is highly probable that strength was positively selected for in men as an important trait in the course of evolution^1,2^.

The extent to which body strength, specifically hand grip strength (HGS), correlates with facial features in men is one of the key issues for evolutionary psychologists, ethologists, and anthropologists. This is particularly relevant in discussions about the mate preferences of heterosexual women. For instance, it is well known that female preferences for more masculine male faces vary across different human populations. Some scholars hypothesize that this variation may be influenced by ecological factors, such as pathogen prevalence, as well as cultural factors^20^, or access to qualified medical care^21^. For example, a study conducted in Turkey found that masculinized faces were perceived as more formidable, slightly healthier, and somewhat more attractive^20^. Similarly, recent findings from African and European samples suggest an association between facial shape, hand grip strength, and facial perception^22–24^.

While patterns of sexual dimorphism vary among populations, the association between hand grip strength (HGS) and facial shape demonstrates cross-population consistency^25–27^. Notably, the link between facial shape and HGS is relatively stable, unidirectional and independent of facial masculinity. Previous research has revealed that facial images of physically strong European men are perceived as more dominant, masculine, and attractive^25^. The geometric morphometric approach proved to be useful in such studies^28^. Based on geometrically morphed images, the components that contribute to (female) perceptions of male physical strength, attractiveness, dominance, and masculinity were identified. Facial features associated with high HGS in European men were: a more robust face with a rounded outline, relatively wider eyebrows, and a more well-curved jaw than in weaker men^29^. A similar pattern of facial morphology associated with HGS was later reported for Maasai^26^, Tuvans of Southern Siberia, and Russians^27^ using strength-calibrated facial morphs. All of the findings mention above are not surprising since, at the proximate level, both HGS and facial traits share a common substrate^23^. That is, genetic, prenatal, and pubertal effects of androgens are supposed to influence the development of facial shape, male muscularity and physical strength^30^. However, other factors moderating this effect also have to be considered^31^.

The data from ‘WEIRD’ (Western, educated, industrialized, rich and democratic) populations^32^ also provide evidence of positive association between physical strength and aggressiveness. However, data on strength-related facial perception in traditional African society (the Maasai of Tanzania)^22^ has raised the question about more differential view of facial images in relation to physical strength, attractiveness, and aggressiveness. It was found that Maasai men and women judged facial morphs calibrated to greater hand grip strength (HGS) higher on strength and attractiveness, but lower on aggressiveness compared to faces calibrated to lesser HGS. Recently, the same strength-calibrated facial morphs were used for assessments by European raters^23^. Response patterns for strength and aggressiveness were similar to those reported for the Maasai intra-population assessments. Facial attractiveness attributions increased with higher HGS in both populations. However, while the image of men with the highest HGS was rated as the most attractive among Maasai, Europeans preferred the image based on medium HGS. Hence, members of the same traditional population (Maasai), and representatives of European ‘WEIRD’ population were able to perceive physical strength from facial morphology properly, and the assessments of aggressiveness were also similar across sexes and populations. Taking into consideration the male-male competition hypothesis, we may suspect a positive evolutionary selection for the association between physical strength and fitness outcomes. Under these conditions, formidable men may have an advantage in competing with other men for resources, status, and partners^10,33^, or signaling fighting qualities through well-developed musculature^34^. Under this perspective, facial shape may serve as a direct cue of formidability^6,26,35^, with young men being especially sensitive to these signals^36,37^. Such visual cues may be generally beneficial in reducing the need for direct physical confrontation between potential rivals.

Human evolution has always been accompanied by active migration and colonization of new territories, highly variable in respect of ecology, including temperature, degree of humidity, seasonality, altitude, pressure of local pathogens and predators. In these dynamic environments, both local human populations and migrating groups pose potential challenges. Under such circumstances, female choice may favor competitive and physically strong men^33,38^, producing a ‘sexy sons’ effect^39^, or otherwise, indirectly, by preference for more dominant and formidable-looking men as sexual partners. Cross-cultural variability in ratings of attractiveness were reported in a number of studies^21,40^, highlighting the importance of population-specific socio-cultural environments, as demonstrated in a recent meta-analysis^41^.

Cooperation and helping group members must have been positively selected in humans^42^. Indirect evidence (studies in modern hunter-gatherers’ societies) suggests that in evolutionary perspective strength could also indicate a better ability to help others. Healthy and strong men and women were better providers, produced more offspring, and offered better resources and protection for their children, thereby enhancing their chances of survival. Recent data from hunter-gatherers, the Hadza of Tanzania, reveal that higher grip strength was associated with better hunting outcomes in men^43^ and was a reliable predictor of helping behavior in women^44^.

The goal of our study was to test cross-cultural variations in perception of young Maasai men composite portraits calibrated to hand grip strength (HGS) by means of geometric morphometrics. We focused on facial judgements on four behavioral traits: physical strength, attractiveness, aggressiveness and helpfulness. Participants of the study were asked to rate three male Maasai portraits (geometric morphometric morphs), which corresponded to low, medium, and high HGS. According to our knowledge, this is the first time, when helpfulness is tested along with three other parameters (physical strength, attractiveness, aggressiveness) in such experimental context. The current study also extends our previous research^22,23^, particularly now we concentrate on the cultural variations among raters.

In order to investigate the breadth of cross-cultural differences, the populations of raters were selected to represent a wide spectrum of people, who differ in their origin, culture and familiarity with African appearance. Raters were recruited from Africa (Tanzania), Europe (Czech Republic and Russia), Asia (Pakistan and China), and Latin America (Mexico), and were representatives of four big human populations: African (Tanzanians), European (Czech, Russians and Pakistani), Asian (Chinese) and Latin American (Mexicans). In addition, our samples differ in relation to marriage norms: Czech, Russians, Mexicans and Chinese practicing monogamy, Pakistani polygynous, and Tanzanians practicing both types of marriage. In Czech, Russian, and Mexican cultures, the choice of a partner is typically left up to the individual’s preference, but it is influenced by relatives’ selection, at least to some extent, in the rest three cultures.

## Results

**Figure 1:**
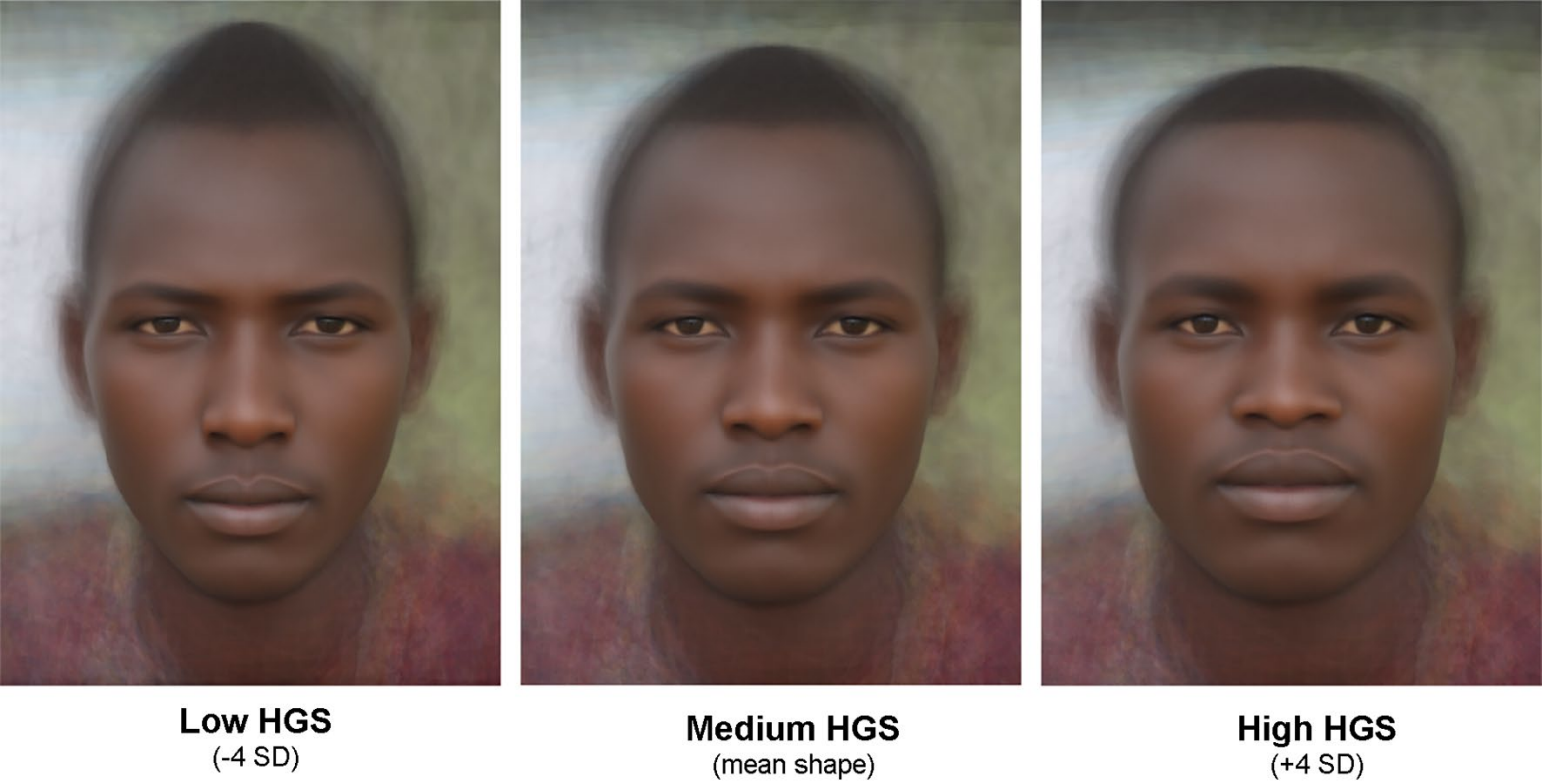
Stimulus portraits of Maasai men representing low, medium and high hand grip strength (HGS). The portraits are averaged and geometrically morphed images. The mean shape (in the middle) corresponds to the Medium HGS; the Low HGS portrait (to the left) represents − 4 standard deviations of HGS from the mean; the high HGS portrait (to the right) represents + 4 standard deviations of HGS from the mean.

The stimulus portraits representing low, medium and high HGS of Maasai men are presented in Fig. 1. For detailed procedure of geometric morphometrics see “Methods” section.

Detailed information on composition of each sample of raters, as well as age differences are presented in Table 1. Since there was notable variation in age between and within samples of raters from different regions (also see Supplementary Table S1), age was controlled for in the main analyses.

**Table 1:** General information about participants and age differences within each of the tested populations.

Population	Men		Women		T	p
	N	Age (M ± SD)	N	Age (M ± SD)		
Tanzanians	103	26.8 ± 6.1	105	26.1 ± 5.2	0.782	0.435
Czechs	79	26.3 ± 9.5	199	23.4 ± 6.7	2.170	0.032
Russians	105	21.1 ± 4.2	235	20.5 ± 4.2	1.195	0.233
Pakistanis	136	26.5 ± 7.0	147	25.5 ± 5.2	1.763	0.174
Chinese	117	32.2 ± 6.3	118	29.2 ± 5.6	3.800	0.001
Mexicans	78	30.3 ± 9.1	118	32.5 ± 10.7	-1.573	0.117
Total	618	27.1 ± 7.9	922	25.3 ± 7.4	4.626	0.001

Age (years)

*M* mean, *SD* standard deviation, *T* Student’s t-test statistics for sex differences in age, *p* statistical significance.

**Figure 2:**
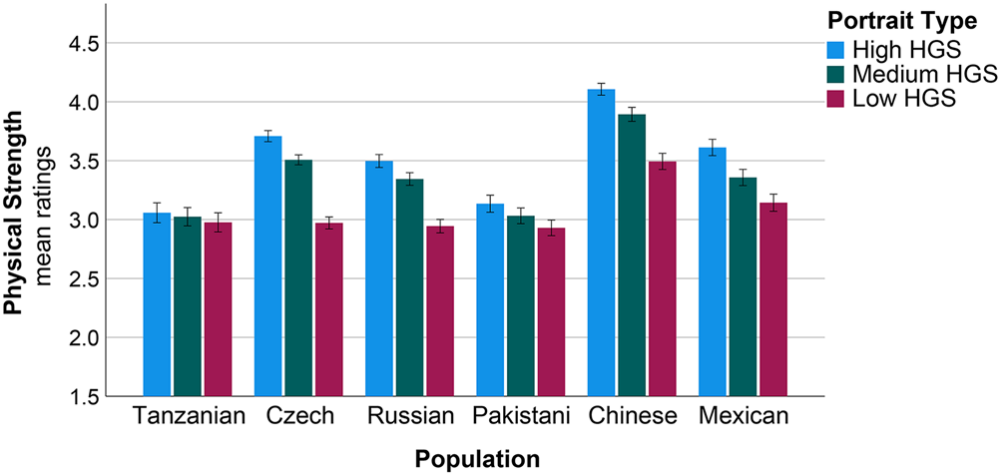
Cross-cultural differences in ratings of physical strength of three young Maasai men portraits based on the average and two extrema of empirical shape variation along the regression vector (± 4 SD of HGS). Ratings varied from 1 to 5. Mean ratings and ± 1 standard error of the mean are presented.

In the case of physical strength as testing variable, the main effects of Age, Sex, Population and portrait type were revealed (Table 2). Generally, older individuals rated all portraits as slightly more physically strong. Men generally provided lower scores on physical strength to all portraits than females. Russians, Czechs, Mexicans, and especially Chinese provided higher scores on physical strength to all portraits (Fig. 2). The main effect of Portrait Type was significant as well (Fig. 2). The morphs of Maasai with low HGS generally received the lowest scores on physical strength. No interaction effect of Population and Sex was revealed. Interaction between Population and Portrait Type was significant. This was mainly due to raters from Czech, Chinese, Russian, and Mexican populations, as only judges from these four populations provided significantly lower scores on physical strength to the “low HGS” portrait (Table 2).

**Table 2:** Effects of sex, population, and portrait type on physical strength ratings.

Dependent variable: physical strength ratings							
Test of model effects				Parameter estimates			
Predictor	X^2^	df	p	Parameter	B	X^2^	p
Age	5.655	1	**0.017**	Age	0.007	5.655	**0.017**
Sex	5.226	1	**0.022**	Female (ref.)			
				Male	− 0.302	9.762	**0.002**
Population	159.98	5	** < 0.001**	Tanzanian (ref.)			
				Pakistani	0.101	0.465	0.495
				Russian	0.308	4.929	**0.026**
				Mexican	0.441	8.822	**0.003**
				Czech	0.546	16.655	** < 0.001**
				Chinese	0.859	41.071	** < 0.001**
Population × Sex	9.265	5	0.099				
Portrait type	214.53	2	** < 0.001**	Low HGS (ref.)			
				Medium HGS	0.383	27.501	** < 0.001**
				High HGS	0.572	42.695	** < 0.001**
Portrait type × Population	69.170	10	** < 0.001**	Low HGS × Czech	− 0.663	40.396	** < 0.001**
				Low HGS × Chinese	− 0.521	22.532	** < 0.001**
				Low HGS × Russians	− 0477	21.333	** < 0.001**
				Low HGS × Mexican	− 0.386	13.558	** < 0.001**
Portrait type × Sex	1.623	2	0.444				

The results of Generalized Estimating Equations for a linear model with repeated measures are presented. Dependent variable—‘physical strength’ ratings, predictors—age, sex, and population of the raters, and the type of portrait [high, medium, or low handgrip strength (HGS)]. Medium HGS corresponds to the mean shape portrait. *X*^*2*^ Wald Chi-Square, *df* degrees of freedom, *B* coefficients, *p* statistical significance (significant effects are highlighted in bold).

**Figure 3:**
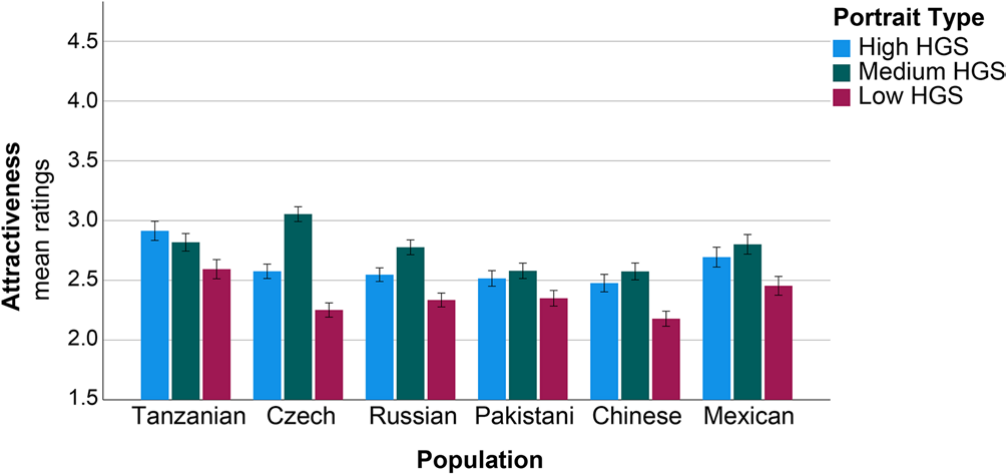
Cross-cultural differences in ratings of attractiveness of three young Maasai men portraits based on the average and two extrema of empirical shape variation along the regression vector (± 4 SD of HGS). Ratings varied from 1 to 5. Mean ratings and ± 1 standard error of the mean are presented.

The ratings on attractiveness revealed no main effects of Age and Sex (Table 3). Whereas, the main effects of Population and Portrait Type were significant. Tanzanians gave the highest scores on attractiveness to all portraits; meantime, Chinese generally provided the lowest scores (Fig. 3). While Tanzanians rated the high HGS portrait as the most attractive, representatives of the rest five populations preferred the portrait of Maasai with medium HGS (mean shape). Universally, the portrait of weak Maasai (low HGS) received the lowest attractiveness scores (Table 3, Fig. 3). The GEE linear model revealed no effect of interaction between population and sex on ratings of attractiveness. Significant interaction effect of Population and Portrait Type was demonstrated, with Czechs and Russians (the only European populations) providing significantly higher scores on attractiveness to the medium HGS portrait (mean shape) (Table 3). The interaction between Portrait Type and Sex was also significant. Generally, females provided higher scores on attractiveness to the medium HGS portrait.

**Table 3:** Effects of sex, population, and portrait type on attractiveness ratings.

Dependent variable: attractiveness ratings							
Test of Model Effects				Parameter Estimates			
Predictor	X^2^	df	p	Parameter	B	X^2^	p
Age	1.114	1	0.286				
Sex	1.796	1	0.180				
Population	29.919	5	** < 0.001**	Tanzanian (ref.)			
				Czech	− 0.167	1.410	0.235
				Mexican	− 0.176	1.308	0.253
				Pakistani	− 0.282	4.538	**0.033**
				Russian	− 0.322	5.393	**0.020**
				Chinese	− 0.457	10.681	** < 0.001**
Population × sex	6.134	5	0.293				
Portrait type	193.75	2	** < 0.001**	Low HGS (ref.)			
				Medium HGS	0.472	32.514	** < 0.001**
				High HGS	0.337	12.808	** < 0.001**
Portrait type × population	65.957	10	** < 0.001**	Medium HGS × Czech	0.559	34.057	** < 0.001**
				Medium HGS × Russian	0.312	11.292	**0.001**
Portrait type × sex	7.196	2	**0.027**	Medium HGS × Males	− 0.153	7.084	**0.008**

The results of Generalized Estimating Equations for a linear model with repeated measures are presented. Dependent variable—‘attractiveness’ ratings, predictors—age, sex, and population of the raters, and the type of portrait [high, medium, or low handgrip strength (HGS)]. Medium HGS corresponds to the mean shape portrait. *X*^*2*^ Wald Chi-Square, *df* degrees of freedom, *B* coefficients, *p* statistical significance (significant effects are highlighted in bold).

**Figure 4:**
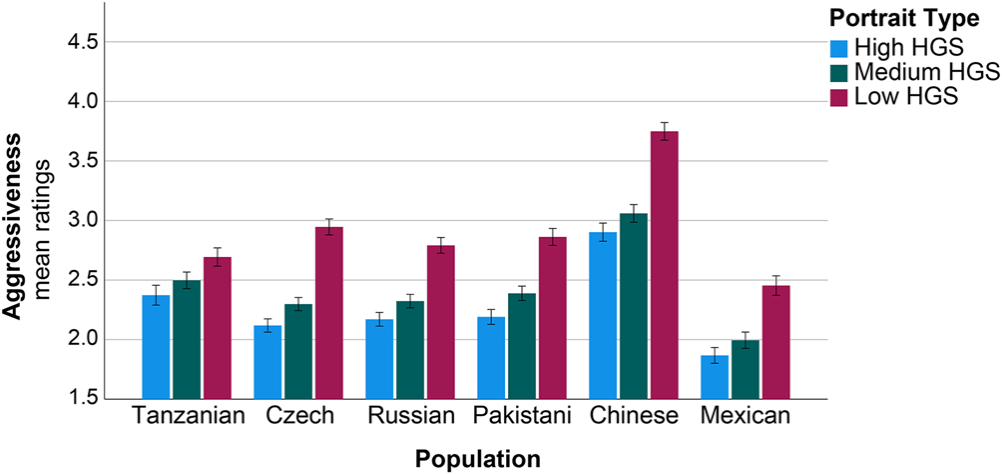
Cross-cultural differences in ratings of aggressiveness of three young Maasai men portraits based on the average and two extrema of empirical shape variation along the regression vector (± 4 SD of HGS). Ratings varied from 1 to 5. Mean ratings and ± 1 standard error of the mean are presented.

The ratings on aggressiveness also revealed no main effect of Age (Table 4). However, in this case, the main effect of Sex was demonstrated. Males generally provided higher scores on aggressiveness to all portraits than females. The main effect of Population was also obtained, with Chinese generally providing the highest scores on aggressiveness to all portraits and Mexicans the lowest (Table 4, Fig. 4). The main effect of Portrait Type was also present. The portrait of weak Maasai (low HGS) generally received the highest scores on aggressiveness (Table 4, Fig. 4). We did not obtain significant interaction between Population and Sex. However, the interaction between Population and Portrait Type was demonstrated. Raters from Mexico, Russia, Pakistan, Czech and China (ordered according to increase of the effect) provided higher scores on aggressiveness to the “low HGS” Maasai portrait compared to Tanzanians (Table 4).

**Table 4:** Effects of sex, population, and portrait type on aggressiveness ratings.

Dependent variable: aggressiveness ratings							
Test of model effects				Parameter estimates			
Predictor	X^2^	df	p	Parameter	B	X^2^	p
Age	0.049	1	0.825				
Sex	10.954	1	** < 0.001**	Male (ref.)			
				Female	− 0.082	0.443	0.506
Population	241.46	5	** < 0.001**	Tanzanian (ref.)			
				Mexican	− 0.365	6.696	**0.010**
				Russian	− 0.181	1.626	0.202
				Pakistani	− 0.108	0.655	0.418
				Czech	− 0.080	0.328	0.567
				Chinese	0.542	14.121	** < 0.001**
Population × sex	4.746	5	0.448				
Portrait type	342.07	2	** < 0.001**	Low HGS (ref.)			
				Medium HGS	− 0.895	66.066	** < 0.001**
				High HGS	− 0.721	57.386	** < 0.001**
Portrait type × population	29.458	10	** < 0.001**	Low HGS × Chinese	0.539	15.231	** < 0.001**
				Low HGS × Czech	0.499	16.929	** < 0.001**
				Low HGS × Pakistani	0.362	8.957	**0.003**
				Low HGS × Russian	0.295	5.960	**0.015**
				Low HGS × Mexican	0.270	4.300	**0.038**
Portrait type × sex	1.800	2	0.407				

The results of Generalized Estimating Equations for a linear model with repeated measures are presented. Dependent variable—‘aggressiveness’ ratings, predictors—age, sex, and population of the raters, and the type of portrait [high, medium, or low handgrip strength (HGS)]. Medium HGS corresponds to the mean shape portrait. *X*^*2*^ Wald Chi-Square, *df* degrees of freedom, *B* coefficients, *p* statistical significance. (significant effects are highlighted in bold).

**Figure 5:**
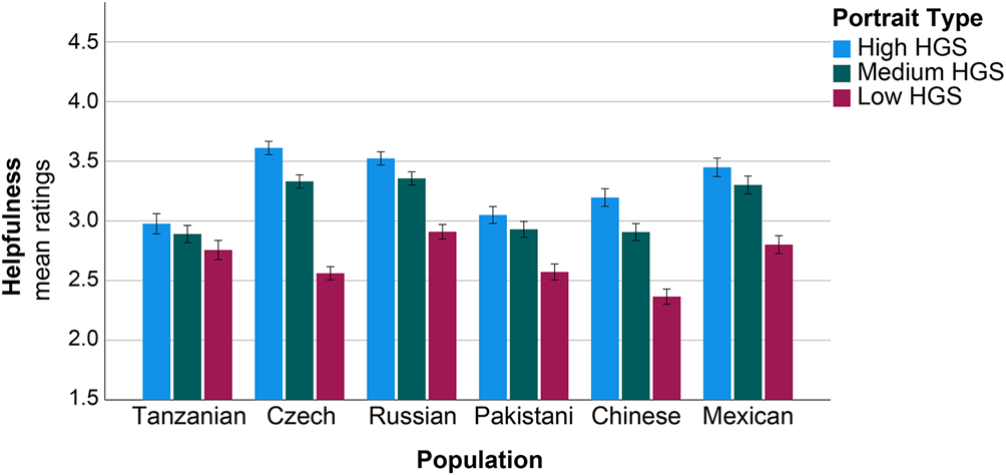
Cross-cultural differences in ratings of helpfulness of three young Maasai men portraits based on the average and two extrema of empirical shape variation along the regression vector (± 4 SD of HGS). Ratings varied from 1 to 5. Mean ratings and ± 1 standard error of the mean are presented.

Finally, the ratings on helpfulness (readiness to help others) were neither associated with Age or Sex (Table 5). The main effect of Population was significant mainly due to Czechs, Russians and Mexicans who generally provided higher scores on readiness to help to all portraits (Table 5, Fig. 5). The main effect of Portrait Type was obtained. The portrait of weak Maasai (low HGS) generally received the lowest scores on readiness to help (Table 5, Fig. 5). There was no interaction effect of Population and Sex. Whereas the interaction between.

**Table 5:** Effects of sex, population, and portrait type on readiness to help ratings.

Dependent variable: readiness to help ratings							
Test of model effects				Parameter estimates			
Predictor	X^2^	df	p	Parameter	B	X^2^	p
Age	0.803	1	0.370				
Sex	1.268	1	0.260				
Population	52.379	5	** < 0.001**	Tanzanian (ref.)			
				Pakistani	0.131	0.878	0.349
				Chinese	0.226	2.436	0.119
				Mexican	0.417	7.242	**0.007**
				Russian	0.427	8.807	**0.003**
				Czech	0.437	9.619	**0.002**
Population × sex	8.127	5	0.149				
Portrait type	384.60	2	** < 0.001**	Low HGS (ref.)			
				Medium HGS	0.542	38.724	** < 0.001**
				High HGS	0.871	77.346	** < 0.001**
Portrait type × population	61.198	10	** < 0.001**	Low HGS × Czech	− 0.797	45.235	** < 0.001**
				Low HGS × Chinese	− 0.594	21.040	** < 0.001**
				Low HGS × Mexican	− 0.404	10.689	**0.001**
				Low HGS × Russian	− 0.364	10.223	**0.001**
				Low HGS × Pakistani	− 0.240	4.152	**0.042**
Portrait Type × sex	2.239	2	0.326				

The results of Generalized Estimating Equations for a linear model with repeated measures are presented. Dependent variable—‘readiness to help’ ratings, predictors—age, sex, and population of the raters, and the type of portrait [high, medium, or low handgrip strength (HGS)]. Medium HGS corresponds to the mean shape portrait. *X*^*2*^ Wald Chi-Square, *df* degrees of freedom, *B* coefficients, *p* statistical significance. (significant effects are highlighted in bold).

Population and Portrait Type was significant. Raters from Czech Republic, China, Russia, and Mexico discriminated significantly between Maasai with high and medium HGS, whereas Tanzanians and Pakistanis did not (Table 5). No interaction between Portrait Type and Sex was observed.

## Discussion

The results of our study revealed that raters from six different cultures were able to distinguish between low, medium, and high hand grip strength (HGS) composite portraits of young Maasai men. Across all six cultures, the ‘weak’ portrait was consistently rated as the least attractive, most aggressive, and least helpful. This suggests that individuals from diverse populations share similar perceptions of physical strength based on facial features. Raters also agreed on attribution of important social qualities such as aggressiveness and helpfulness to composite portraits.

The results of our cross-cultural study extend our understanding of universal basis of perception of human appearance, particularly, facial cues of physical and behavioural qualities. At the same time, it reveals culturally specific differences in ratings of these qualities. Participants from Sub-Saharan African, Slavic-Orthodox, Euro-Western, Islamic, East Asian, and Latin American cultural regions, who represented four big human populations (Africans, Europeans, Asians, and Latin Americans, accordingly) were able to differentiate between physically strong and weak portraits of young Maasai men. Particularly, the image with low HGS received the lowest scores on physical strength in all cases. Hence, the previous results obtained from the Maasai themselves and Europeans^22,23^ were confirmed on much broader cross-cultural sample. We conclude that, independent of the degree of familiarity with representatives of a particular population (distinct both in respect of racial and cultural characters), humans are able to discriminate properly between portraits of strong and weak men. These results provide empirical support for the idea of positive selection favoring stronger men in human evolution^1,2,9,10^.

Still, we believe some minor cross-cultural differences deserve special attention. Our study revealed that only Tanzanian raters selected the composite portrait of men with high hand grip strength (HGS) as the most attractive. In contrast, in the other samples, both high and medium HGS images were rated as attractive. Strong men historically had advantages in environments with high pathogen stress, male-male conflicts, social status, and mate selection, potentially leading to increased offspring in our evolutionary past^8,10^. This preference for strength may still hold value in Tanzania, where good genes could be crucial for offspring survival. However, in the other five populations, a shift towards selecting good fathers may be increasingly beneficial. One should keep in mind that Tanzania was the only country among the six on our list that belonged to the group of least developed countries according to the UN 2023 classification^45^.

By demonstrating that the low HGS face was universally rated as the most aggressive, we extend the list of recent facial perception studies revealing that more formidable men are not rated as the most aggressive, just the opposite, weaker young men were selected as more aggressive (see^22–24^). Indeed, across all six samples, the low HGS portrait consistently received the highest aggression ratings, with universally significant differences compared to the medium and high HGS portraits. Regardless of whether raters came from populations with generally low or high physical strength^15^, aggressiveness was attributed to the weaker composite image. Our results, along with others mentioned above, challenge some previous research on facial cues related to dominance, formidability, and fighting potential^36,37^. One should be very careful in this respect as appearing formidable does not necessarily equate to being more aggressive.

Our results also pointed to the effects of Age, Sex and Population in the perception of physical strength. Older individuals were generally inclined to rate all portraits as slightly more physically strong. This may be due to personal life experiences that suggest younger men to be generally stronger than older men. This assertion is supported by numerous reports from different postindustrial as well as traditional populations confirming that physical strength declines with age^46–51^. It was demonstrated that men generally scored physical strength of young men images lower compared to women. We suggest that this may reflect widely spread gender stereotypes, where women generally feel men (especially young men) as being stronger than themselves, while men may seek to downplay the qualities of other men^52,53^.

Differences in familiarity with representatives of other distinct populations, along with stereotype images of distinct others may partly explain the general differences in ratings of physical strength, particularly, much higher scores on physical strength given by Chinese participants. Facial features of the Maasai are more similar to those of Czechs, Russians, and Pakistanis, and differ more from Chinese (Han), with Mexicans falling in between. Migration patterns also play a role, with people of African origin being more common in Europe and Latin America compared to China^54^. Another important issue worth mentioning here is the general differences in mean and/or median HGS of young men across populations from developed and developing regions, as suggested by meta-analysis conducted by Dodds with co-authors^15^. Chinese individuals tend to have lower HGS compared to Czechs or Russians, for example^55–57^.

In our study, ratings of morphed Maasai portraits on attractiveness were not related to Age and Sex of respondents. However, the main effect of Population was significant. Tanzanians gave the highest scores on attractiveness in general, and Chinese, on the contrary, provided the lowest scores. This difference could be attributed to familiarity with certain appearances and the resemblance of the morphed portraits to the respondents’ own population features (as mentioned above). Skin color, in addition to facial shape, may also play a role in these ratings. It is worth noting that Chinese individuals generally prefer lighter skin in partners and perceive white skin as more attractive^58,59^, healthier, and younger-looking^60^. This preference contrasts with Europeans, who do not prioritize white skin in the same way^59,60^. Tanzanians rated the Maasai portrait with high HGS as the most attractive, aligning with preferences of the Maasai raters themselves, obtained in our previous study^22^. The ratings on attractiveness received from subjects of other populations were consistent with recent findings obtained among European students, where the portrait with medium HGS (mean shape) received the highest scores^23^. Again, it is noteworthy that representatives of European populations (Russians and Czechs) provided significantly higher scores on attractiveness to the medium HGS portrait, compared to Mexicans, Pakistanis, and Chinese. Our findings provide more fuel for discussion about cultural variations in preferences for sex-typical traits (physical strength in our study)^21,61,62^. Our data revealed that raters from Tanzanian population considered the high HGS morph as the most attractive, while raters from other populations preferred the portrait of men with medium HGS (mean shape portrait). Hence, our findings are rather in line with data obtained by DeBruine with colleagues^21^. In their study, they suggested that preferences for more masculinized male faces are positively correlated with high pathogens level and limited access to medical care. Interestingly, according to the health index score, based on health and health systems ranking of countries worldwide in 2023, Tanzania was rated lowest in our list of six countries with the index score of 60.1, and China being the other pole with the score of 83.1, the rest of countries being in between^63^.

Our analysis of aggressiveness ratings revealed significant effects of Population and Portrait Type. Chinese participants (the most distinct population, the least experienced with Africans) provided the highest scores on aggressiveness to all portraits. This may suggest that lack of familiarity with representatives of other population may stimulate suspicion and negative feelings. The main effect of Portrait Type was also demonstrated, and the portrait of weak Maasai (low HGS) generally received the highest scores on aggressiveness. Hence, our current cross-cultural study confirms the earlier findings obtained among Maasai and Europeans^22,23^. The portrait with low HGS, rather than high HGS was rated as more aggressive.

There was no significant effect of Age on aggressiveness ratings, however, the main effect of Sex was significant. Males generally provided higher scores on aggressiveness to all portraits compared to females. As discussed above, males also rated all portraits lower on physical strength than females. This may provide an interesting insight into male-male perception. Scoring other men lower on physical strength and higher on aggressiveness may reflect competitive motivations: an orientation towards a potential threat and, at the same time, an increase of the fear threshold. Such perception strategy may be adaptive in the evolutionary past, given numerous cases of intergroup conflicts all over the world. This result is also related to one of the main findings of our present and earlier studies, namely, that perception of male physical strength and aggressiveness based on facial cues are negatively correlated. This effect is evident here in the form of the sex differences: females on average perceived men as physically stronger and less aggressive, while men perceived other men as less physically strong and more aggressive. This finding may reflect a deeply imbedded negative link between physical strength and perceived aggressiveness.

It is widely accepted that human male mating and reproductive adaptations are more associated with dominance and status, while female adaptations are strongly linked with signals of reproductive quality and health to attract mates^64^. The association between facial shape, body strength, aggressiveness, and dominance in human males was also claimed to be universal^65,66^. However, the newly accumulated data suggest that associations between facial shape, aggression, and dominance may be much more complicated. Findings from a recent study (^37^: 144) suggest that “when two sexually dimorphic androgen dependent facial traits are judged in concert, ornamental rather than structural masculine facial features underpin men’s intra-sexual judgments of formidability”. Taking into consideration all these findings, we conclude that the widely present believes on the association between facial shape and body strength with aggressiveness and dominance in human males^65,66^ have to be taken at least with limitations.

This is the first time that the male facial morphs based on HGS were subjected to ratings on helpfulness. Age and sex did not influence helpfulness ratings, but there was a significant effect of Population. Czechs, Russians, and Mexicans were the most “sympathetic” towards all images and provided generally higher scores on readiness to help to all portraits. It is important to stress that respondents from all six populations rated the portrait of men with low HGS as the lowest in helping potential. Hence, we conclude that irrespective of cultural and racial differences, people associate facial traits of weaker men with lower ability to help others. Czechs, Chinese, Russians, and Mexicans attributed significantly higher ratings on helpfulness to the Maasai portrait with high HGS compared to medium HGS, whereas Tanzanians and Pakistanis did not. Our study is the first to assess male facial characteristics based on hand grip strength (HGS) in terms of helpfulness ratings. Further research is needed to understand why certain populations may be less discriminative in assessing potential helpfulness based on physical traits. It is possible that in communalistic societies like those in Africa or Pakistan^67,68^, distinguishing between individuals who can provide help and those who cannot is of paramount importance.

Our study has certain limitations. The portrait ratings were implemented in four different languages (English, Spanish, Russian, and Chinese). We acknowledge that some cultural differences (e.g. atypically high scores on aggressiveness provided by Chinese participants for all portraits) could be partly explained by variations in how words that denote the rated traits are interpreted in different languages. For example, the term ‘aggression’ has multiple meanings in English. Translating it into different languages might introduce some variation based on subtle connotations and cultural specificity. Future studies should consider this issue more thoroughly, possibly by offering a few options of discriminant versions of certain behavior, aggression in particular. Another limitation is that facial shape, which signals physical strength, may also convey information about other qualities related to physical strength that were not considered in our study. These additional qualities, such as physical, communicative, or even personality traits, could introduce some noise in reactions (ratings) and their interpretations. However, this limitation generally applies to all studies of facial perception.

In summary, our study confirms the expectation that adult individuals, both men and women, are able to distinguish between physically strong and weak images of men, irrespective of whether raters are estimating photos of individuals from their own or highly distinctive human population both in terms of anthropometric and cultural features. Respondents from different populations were generally in consensus on attributing aggressiveness and helpfulness to facial images of men with low, medium, and high hand grip strength. While the portrait of weaker men was universally treated as unattractive, preferences for images of high and medium HGS men varied across cultures. We propose that the observed differences between populations were influenced by environmental factors (e.g., high pathogen levels), culture-specific demands, and economic conditions (including access to healthcare and its quality), as well as cultural stereotypes of attractiveness for men and women, which are increasingly shaped by mass media and global advertising in the modern world.

## Methods

### Data collection and participants

Participants from Africa, Europe, Asia, and America represented Cultural Regions: Sub-Saharan African, Euro-Western, Slavic-Orthodox, Islamic, East Asian, and Latin American^69^, and four big human populations, accordingly (Africans, Europeans, Asians, Latin Americans). Raters were men and women from six countries: Tanzania, Czech Republic, Russia, Pakistan, China, and Mexico. In total, there were 1540 raters in our sample, 618 men and 922 women. The mean age of participants was 26.0 ± 7.6 years, ranging from 17 to 50 years. Refer to Table 1 for more details on each sample. The samples varied significantly by age (F = 97.21, df = 5, p = 0.001). Russians were the youngest, Chinese the oldest among the populations. More information is presented in Supplementary Table S1.

Tanzanians represented a population of African descent, and consisted predominantly of Bantu ethnic groups (Chagga, Gogo, Hehe, Haya, Ngoni, Nyamwezi, Sukuma, Zigua, and others). The main religious affiliations were Christianity and Islam. In the country, 55.3% of population identify themselves as Christians and 31.5% as Islamic^70^. All of the Tanzanian participants were English-speaking. English language was used for data collection.

The Czech sample was collected in Ostrava city, represented by Czechs who identified themselves as Christians of predominantly Catholics nomination or Atheists. According to general information, 47.8% of Czechs identify themselves as agnostics^71^. Czech language was used for data collection.

The Russian sample was represented by Russians from Tula city. Majority were Orthodox Christians. According to general information, 47.4% of Russian population identify themselves as Christians^72^. Russian language was used for data collection.

The Pakistan sample was collected in Haripur, represented by Pakistanis. All identified as Islamic. According to general information, 96.5% of Pakistanis are Islamic^73^. English language was used for data collection.

Chinese sample was predominantly represented by Han Chinese (97%) from 98 diverse cities across China. Most of them were Atheists, with few identifying themselves as Buddhists and Taoists. According to official statistics, 73.6% of population in China are agnostics^74^. Chinese language was used for data collection.

Mexican sample was mainly collected in the center of the country: the states of Veracruz, Puebla and Mexico City. Mestizos of mixed Native American, European and African descent are a predominant ethnic group in contemporary Mexico, although many Mexicans do not identify as Mestizos; a small fraction of the sample was represented by indigenous groups (Nahuas, Otomis, Tepehuas, Totonacs, etc.). The country’s dominant religious affiliation is Roman Catholicism, 91,3% of population are Christians in total^75^. The participants were all Spanish-speaking. Spanish language was used for data collection.

Three samples are of European origin (Czechs, Russians and Pakistanis), one of African (Tanzanians), one Asian (Chinese), and one is of mix descent (Mexicans). Based on genetic distance data, Asians and Africans are more distinct from each other than Europeans and Asians, or Europeans and Africans^76,77^. Using estimated differences in genetic variation (measured by genetic distance as a molecular clock in evolutionary trees), the information about the time of population separations may be created (traditional method); besides, the new methods based on case–control association studies may be used^78–80^. In all cases, the Sub-Saharan African populations (Maasai being one of them) are more distinct from Han (Chinese) then from Europeans (in our case, Czechs, Russians, and Pakistanis). As for contemporary Mexicans, their genetic ancestries have been traced to Western Europe, West Africa, and East Asia, and could be identified: 73.8% of Indigenous, 19.8% of European, 5.2% of African, and 0.8% of East Asian ancestries in the case of the State of Veracruz with similar scores for the state of Puebla and Mexico City (^81^: 777). It is also mentioned that Mesoamerican indigenous populations are closer related to Han, less to North Eurasian and even less to African ones genetically^82,83^.

Familiarity with the African appearance is influenced by historical and modern international migration flows from Sub-Saharan Africa^54^. Recent data shows that the primary migration destinations from Africa are still the USA (often involving transit through Latin America) and Europe, with minimal migration to China. These data suggest that European and Latin American populations are likely to have the highest familiarity with African appearance in our study, while the Chinese population may have the least exposure to it.

The study was conducted in compliance with ethical standards and the Declaration of Helsinki on Biomedical Research Involving Human Subjects. The study was approved by the Institutional Review Board of the Institute of Ethnology and Anthropology of the Russian Academy of Sciences (Protocol No: 005; 16.02.2023). Informed consent was obtained from all subjects, and their anonymity was ensured.

### Stimuli

The stimulus portraits for cross-cultural judgements were created by means of geometric morphometrics^84,85^. The calibration sample comprised images of 54 young-adult Maasai men (20–29 years of age) from the Ngorongoro area of Northern Tanzania. Their facial photographs were taken in full-face perspective, with head positioned according to the Frankfort horizontal plane. Seventy-one facial landmarks and semilandmarks were then placed on each photograph in tpsDig2 2.17^86^. The detailed information on the procedure of landmarks’ digitalization and facial shape configuration are reported in the earlier studies^26,29^. According to the geometric morphometric methodology ^87^, all facial configurations were standardized by Generalized Procrustes superimposition. This was done together with sliding semilandmarks using minimum bending energy criterion in “geomorph” package for R^88^. Since fluctuating asymmetry was not within the scope of this study, in order to reduce possible shape distortion, facial configurations were also symmetrized using basic R functions, and those developed by Claude^89^.

The hand grip strength was measured directly (in kgf) using portable hand dynamometer (DMER-120, Tulinovsky Instruments, Tulinokva, Russia). Subsequently, the facial shape configurations were regressed upon HGS by multivariate regression in tpsRegr 1.45^86^. The fitted model was used to find facial shape configurations corresponding to mean, low (− 4 SD) and high (+ 4 SD) HGS. The stimulus portraits were created by unwarping and averaging photographs towards target configurations in tpsSuper 2.04^86^. Eventually, the stimulus portraits represented not just composites, but the composites morphed according to the fitted regression model, capturing the vector of facial shape deformation based on the HGS. In this study, we restricted stimuli set to three morphs. This was done to provide the respondents with more distinct variations between presented stimuli, given the highly variable contingent of raters both in terms of population and cultural origin.

### Rating study

The subjects of our study were recruited by spreading a web-link to the online study form. Participants were asked to judge the geometric morphometric morphs of young Maasai men representing the sample average and two extrema of empirical shape variation along the regression vector (± 4 SD of HGS). The ratings were provided on traits: ‘physically strong’, ‘aggressive’, ‘ready to help’, and ‘attractive’ using the 5-point scale (namely, ‘not at all’, ‘not much’, ‘moderately’, ‘somewhat yes’, ‘very much’). The order of presentation of the three morphs, and the order of judged traits were fully randomized. In addition, the online form contained questions about age, sex, place of birth, ethnic origin, and religion of participants.

### Statistical analysis

To assess how ratings of the portraits were affected by the type of a portrait (low, medium, or high HGS morph), as well as by the raters’ age, sex, and population origin, the Generalized Estimating Equations (GEE) as linear models with repeated measures were used (N_measures per subject_ = 3). Ratings on each trait were analysed separately. In each model, ratings of the portraits were set as a response variable, and portrait type, raters’ age, sex, and population origin, as well as their interactions, were set as predictors. The analysis was performed in IBM SPSS 27.

### Supplementary Information

Supplementary Information.

## Data Availability

The datasets presented in this study can be found at: FigShare.com, the data are available at 10.6084/m9.figshare.25197476.
