# Supplementary material for: Cross-cultural perception of strength, attractiveness, aggressiveness and helpfulness of Maasai male faces calibrated to handgrip strength
Source: Sci Rep. 2024 Mar 11;14:5880. doi: 10.1038/s41598-024-56607-z (PMC10928163; doi:10.1038/s41598-024-56607-z)
Supplement: Supplementary file 1 — Supplementary Information. [file 41598_2024_56607_MOESM1_ESM.pdf]

Supplementary Materials

Supplementary Table S1. Post Hoc Tests (Bonferroni) on multiple comparisons on age differences between participants from six populations

Multiple Comparisons						
Dependent Variable: Age						
Bonferroni						
(I) Population	(J) Population	Mean Difference (I-J)	Std. Error	Sig.	95% Confidence Interval	
					Lower Bound	Upper Bound
Tanzanian	Pakistani	.491	.609	1.000	-1.30	2.28
	Mexican	-5.170*	.664	<.001	-7.12	-3.22
	Czech	1.966*	.611	.020	.17	3.76
	Russian	5.755*	.587	<.001	4.03	7.48
	Chinese	-4.235*	.635	<.001	-6.10	-2.37
Pakistani	Tanzanian	-.491	.609	1.000	-2.28	1.30
	Mexican	-5.660*	.619	<.001	-7.48	-3.84
	Czech	1.476	.563	.132	-.18	3.13
	Russian	5.265*	.536	<.001	3.69	6.84
	Chinese	-4.725*	.588	<.001	-6.45	-3.00
Mexican	Tanzanian	5.170*	.664	<.001	3.22	7.12
	Pakistani	5.660*	.619	<.001	3.84	7.48
	Czech	7.136*	.622	<.001	5.31	8.96
	Russian	10.925*	.598	<.001	9.17	12.68
	Chinese	.935	.645	1.000	-.96	2.83
Czech	Tanzanian	-1.966*	.611	.020	-3.76	-.17
	Pakistani	-1.476	.563	.132	-3.13	.18
	Mexican	-7.136*	.622	<.001	-8.96	-5.31
	Russian	3.789*	.539	<.001	2.20	5.37
	Chinese	-6.201*	.591	<.001	-7.94	-4.46
Russian	Tanzanian	-5.755*	.587	<.001	-7.48	-4.03
	Pakistani	-5.265*	.536	<.001	-6.84	-3.69
	Mexican	-10.925*	.598	<.001	-12.68	-9.17
	Czech	-3.789*	.539	<.001	-5.37	-2.20
	Chinese	-9.990*	.565	<.001	-11.65	-8.33
Chinese	Tanzanian	4.235*	.635	<.001	2.37	6.10
	Pakistani	4.725*	.588	<.001	3.00	6.45
	Mexican	-.935	.645	1.000	-2.83	.96
	Czech	6.201*	.591	<.001	4.46	7.94
	Russian	9.990*	.565	<.001	8.33	11.65

Sig. - statistical significance (p); * p <.05.
